# Theta Frequency Electromagnetic Stimulation Enhances Functional Recovery After Stroke

**DOI:** 10.1007/s12975-023-01202-z

**Published:** 2023-11-14

**Authors:** Naohiko Okabe, Mary Hovanesyan, Srbui Azarapetian, Weiye Dai, Batsheva Weisinger, Ana Parabucki, Shira Reznik Balter, Esther Shohami, Yaron Segal, S. Thomas Carmichael

**Affiliations:** 1 Department of Neurology, David Geffen School of Medicine, UCLA, Los Angeles, CA 90095 USA; 2 BrainQ Technologies, Ltd., Jerusalem, Israel; 3 Hebrew University of Jerusalem, Jerusalem, Israel

**Keywords:** Stroke, Extremely low-frequency low-intensity electromagnetic field therapy, Motor function, Plasticity

## Abstract

Extremely low-frequency, low-intensity electromagnetic field (ELF-EMF) therapy is a non-invasive brain stimulation method that can modulate neuroprotection and neuroplasticity. ELF-EMF was recently shown to enhance recovery in human stroke in a small pilot clinical trial (NCT04039178). ELF-EMFs encompass a wide range of frequencies, typically ranging from 1 to 100 Hz, and their effects can vary depending on the specific frequency employed. However, whether and to what extent the effectiveness of ELF-EMFs depends on the frequency remains unclear. In the present study, we aimed to assess the efficacy of different frequency-intensity protocols of ELF-EMF in promoting functional recovery in a mouse cortical stroke model with treatment initiated 4 days after the stroke, employing a series of motor behavior tests. Our findings demonstrate that a theta-frequency ELF-EMF (5 Hz) effectively enhances functional recovery in a reach-to-grasp task, whereas neither gamma-frequency (40 Hz) nor combination frequency (5–16-40 Hz) ELF-EMFs induce a significant effect. Importantly, our histological analysis reveals that none of the ELF-EMF protocols employed in our study affect infarct volume, inflammatory, or glial activation, suggesting that the observed beneficial effects may be mediated through non-neuroprotective mechanisms. Our data indicate that ELF-EMFs have an influence on functional recovery after stroke, and this effect is contingent upon the specific frequency used. These findings underscore the critical importance of optimizing the protocol parameters to maximize the beneficial effects of ELF-EMF. Further research is warranted to elucidate the underlying mechanisms and refine the protocol parameters for optimal therapeutic outcomes in stroke rehabilitation.

## Introduction

The impairment of upper extremity function is a common and significant issue experienced by the majority of stroke patients [1–3]. Despite the potential for rehabilitative training to improve functional outcomes, several factors, including stroke severity [4], age [5], comorbidity [6–8], and limited access to rehabilitation services [9], often hinder the achievement of optimal recovery. To address this challenge and enhance functional recovery, various non-invasive brain stimulation techniques, such as repetitive transcranial magnetic stimulation (rTMS), transcranial direct current stimulation (tDCS), and vagal nerve stimulation (VNS), have shown promising results in clinical trials [10–12]. However, these approaches have not been widely adopted, possibly due to factors such as device availability and inconsistent or limited beneficial effects [10, 11].

Among the diverse range of non-invasive brain stimulation techniques, extremely low-frequency, low-intensity electromagnetic fields (ELF-EMFs) have emerged as relatively safe and accessible therapeutic option, which demands further exploration. Although ELF-EMFs do not directly induce action potentials, a growing body of evidence indicates their ability to modulate various biological events, including inflammation [12, 13], oxidative stress response [14–16], apoptosis [17], neurogenesis [18–20], axon outgrowth and synaptogenesis [21], and neuron-astrocyte synchronization [22] by targeting diverse signaling pathways such as calcium signaling [17, 20, 22–25], nitric oxide signaling [10, 26–28], adenosine receptors [13, 29] and magnetoreceptors [16, 21]. ELF-EMFs have also demonstrated neuroprotective and neurorestorative effects in both preclinical stroke models [18, 26, 30–32] and clinical studies with stroke patients [27, 33–35]. For example, exposure to ELF-EMF in a frequency-specific manner utilizing motor-related oscillations (Electromagnetic Network Targeting Field: ENTF) resulted in significant improvement in upper extremity motor function and reduced overall disability in a subacute ischemic stroke population, suggesting its potential as a viable post-stroke therapy [33].

The effect of electromagnetic stimulation can vary depending on the intensity [36] and frequency [21, 37, 38] of the applied stimulation. However, the effects of ELF-EMFs with different intensity and frequency protocols have not been systematically investigated, especially with respect to functional recovery in the subacute phase when little neuronal cell death occurs. Given the current stroke management in which recanalization therapy is the gold standard, noninvasive brain stimulation therapy is expected to serve as an adjunctive therapy when recanalization therapy is not possible. Therefore, optimizing ELF-EMF stimulation parameters for the post-stroke recovery in the subacute phase is critical to enhance the utility of this therapy.

This experiment aims to discern whether various frequencies and intensities of ELF-EMF stimulation have unique effects on functional recovery in the subacute phase after stroke, specifically focusing on forelimb motor function. Frequencies were selected for their known association with motor function and/or neuroplastic recovery techniques in electroencephalogram studies. Most significantly, gamma-band oscillations in general, and 40 Hz in specific, are important for learning and memory and for setting how the brain will form new connections as it develops. It has been demonstrated that invasive entrainment of such oscillatory patterns can induce a renewed plasticity state in mature animals [39]. Human and rodent studies also showed enhanced theta coherence, typically 4 to 12 Hz in mice, to be correlated with high cognitive effort or performance [40, 41]. Additionally, in rodents, this activity range has been linked to sniffing and sensorimotor performance [42]. Furthermore, rTMS at 5 Hz has shown sustained improvement in motor function and disability following stroke [43]. Finally, among possible coherence frequencies associated with stroke recovery or motor control, 16 Hz is a representative frequency from the beta range, which is established as being important for motor planning [44]. While the main objective of the study was to identify parameter-specific responses to ELF-EMF stimulation, there was an additional interest in assessing the effect of stimulations more similar to endogenous brain activity. As neuronal signals are comprised of more complex waveforms rather than isolated individual wavelengths, a combinatory condition with a representative frequency from each band of interest (beta, gamma, and theta) was also tested. These conditions, 5, 16, and 40 Hz stimulation were applied serially for 40 min each, for a total of 120 min as with the single frequency conditions. Effects in this condition may provide insight into possible combinatory effects that would not be seen in the single-frequency conditions. This would also test, to some extent, the time dependency of the treatment effect. Regarding treatment intensity, two intensities were chosen. These intensities were chosen keeping clinical translation in mind, and are based on previously successful clinical studies [33], or otherwise within the range established as safe and effective for human exposure [45].

In the present study, we induced photothrombotic stroke in mice and assessed the effect on motor function of five different ELF-EMF protocols, varying stimulation intensity (measured in Gauss, G) and frequency: 2.5G/5–12-40 Hz, 0.35G/5–12-40 Hz, 2.5G/40 Hz, 0.35G/40 Hz, and 0.35G/5 Hz. These protocols were compared to a sham stimulation protocol (0G/0 Hz). ELF-EMF was initiated 4 days after the stroke induction, a time period that is clinically relevant for a neural repair therapy, and in which neuronal cell death and injury have subsided [46]. We performed a series of motor tests at various time points to comprehensively evaluate forelimb motor function restoration. Additionally, we employed histological analysis for infarct volume and neuroinflammation to gain insights into the biological mechanisms underlying the observed functional changes resulting from the ELF-EMF treatments.

## Methods

### Mice

A total of 72 (12 mice/group) 10-week-old C57BL/6 J (Jackson Laboratories) mice were used in this study. All mice were housed in a temperature-controlled vivarium that ran on a 12-h light/dark cycle. Food was monitored and restricted at times during the study prior to pasta matrix training. All experiments were performed in accordance with the National Institutes of Health animal protection guidelines and were approved by the University of California, Los Angeles Chancellor’s Animal Research Committee.

### Experimental Design

**Fig. 1:**
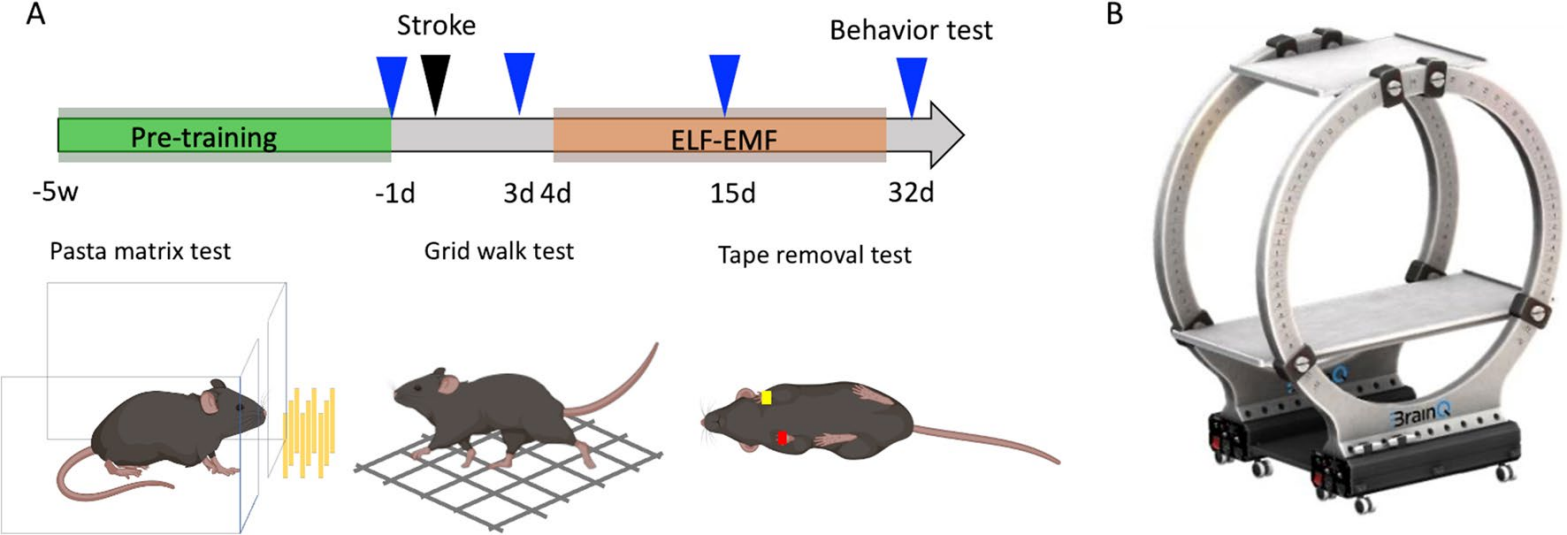
Experimental design. **A** The mice received motor task acquisition training for the pasta matrix test for 5 weeks before the photothrombotic stroke (PT). Behavior tests were conducted pre and 3, 15, and 32 days after the stroke. ELF-EMF treatment or sham was initiated 4 days after stroke. Blue arrowheads indicate behavior tests; black arrowheads indicate stroke surgery. **B** ELF-EMF device. The housing cages were placed on the acrylic tray between the two EMF-generating coils. The treatment protocols were number-coded, and the blinded experimenter conducted the treatment. The device does not generate sound or vibration to alert the experimenter to the fact that it emits the field, nor was any behavioral differences between the groups once the device was emitting the field or not, and therefore the experimenter could stay blinded the whole experiment

Mice were randomly divided into 6 groups: 2.5G/5–12-40 Hz, 0.35G/5–12-40 Hz, 2.5G/40 Hz, 0.35G/40 Hz, 0.35G/5 Hz, and 0G/0 Hz (Sham stimulation): *n* = 12 / group. We determined the sample size based on the previous studies in our lab that demonstrated this number of animals is enough to assess the effects on behavioral recovery after a stroke [47–49]. The stimulation parameters used were selected aligning with findings from successful clinical studies. We conducted three motor tests to assess both learned motor functions (pasta matrix test) and innate motor functions (grid walk and tape removal test). For the pasta matrix test, mice received motor task acquisition training for 5 weeks before the stroke induction. After completion of acquisition training for the pasta-matrix test, mice underwent photothrombotic stroke (PT) in the forelimb area of the primary motor cortex in the left hemisphere. Behavior tests were conducted 1 day before the stroke, and 3 days, 15 days, and 32 days after the stroke. Since the pasta matrix test is sensitive to environmental changes, we omitted the pasta matrix test 15 days after the stroke, in which the ELF-EMFs treatment occupied a regular testing timeframe (Fig. 1A). Mice were number-coded, and all experimental procedures, including stroke surgery, ELF-EMF treatment, behavioral testing and analysis, histological analysis, and statistical testing, were performed in a blind fashion. ELF-EMF treatment or sham was begun 4 days after stroke. For the GFAP and Iba1 histological analyses, four age-matched naïve mice were added as control to validate the effect of stroke.

### Electromagnetic Stimulation

The ELF-EMF device consisted of a 56-turn Helmholtz coils (42 cm radius), capable of generating 1–100 Hz EMFs at intensities of 0.3 to 10G (Fig. 1B). A Helmholtz setup was chosen specifically for its relatively large region of field homogeneity, which was established both mathematically, and confirmed via significant quality testing as having less than 5% variability. EMFs were produced by a BK Precision 4045B sine wave generator and amplified by a built-in amplifier. The field that the device is generating covers the whole brain and is not directed to a specific location, which means that it stimulates neural networks wherever they exist in the insulted brain. Mice behaved freely within their home cage placed on an acrylic platform centered between the coils, such that the entire cage was situated within a homogeneous EMF, without restriction to specific body or brain orientation, exposing all animals to an identical field. Metal components, including wire mesh food holder and water bottle nozzle, were kept inside the cages since these metal components do not influence EMF delivery. The strength and stability of the field were monitored throughout exposure sessions by an ELF-EMF detector probe and Gauss meter. After ELF-EMF exposure, mice were returned to their housing environment.

ELF-EMF exposure was for 120 min daily for each experimental group. Experimental groups manipulated both the frequency (5 Hz, 40 Hz, and a combination of 5–16-40 Hz) and intensity (0.35 G and 2.5 G) of stimulation, with the sham control group being placed in the device with the field set to 0 Hz and 0 G (Table 1). The experimenter operating the device knew only the group number, not the experimental condition, and ran the treatment program with the same protocol number as the group number. We started ELF-EMF treatment 4 days after the stroke, timed for the subacute period in which stroke recovery occurs, and continued for 4 weeks, 5 days a week.

**Table 1:** Frequency and intensity parameters for the ELF-EMF protocols

Group	Frequency	Intensity
1	5–16-40 Hz	2.5 G
2	5–16-40 Hz	0.35 G
3	40 Hz	2.5 G
4	40 Hz	0.35 G
5	5 Hz	0.35 G
6 (control)	0 Hz	0 G

### Photothrombotic Stroke

Photothrombotic stroke was induced, as described previously as it allows precise lesioning [50]. In the present study, photothrombotic stroke was made targeting the caudal forelimb area of the primary motor cortex. We previously demonstrated that photothrombotic stroke targeting the CFA induces neuronal plasticity, including changes in motor map representation [51] and axonal sprouting [49] in the remaining motor areas. Mice were anesthetized with 2% isoflurane, positioned in a stereotaxic instrument, and intraperitoneally administered with Rose Bengal (1% saline, 0.01 mL/g). Five minutes after the Rose Bengal administration, the skull over the forelimb area of the primary motor area (1.5 mm lateral, 0.25 mm rostral to the bregma) was illuminated with a 2 mm diameter green light (Thorlabs) at 20 mW for 10 min. This coordinate and size of illumination targets a large part of the caudal forelimb area in the primary motor cortex but preserves the rest of motor and sensory cortices [52]. The rectal temperature of the mice was maintained at 37 ± 0.5 °C throughout the surgery. Two percent Marcaine was applied under wound margins at the end of the procedure for pain management.

### Behavioral Assay

#### Pasta Matrix Test

We conducted the pasta matrix test to assess learned motor performance as described with slight modifications [53]. All mice were trained for 5 weeks (5 days/week, Monday to Friday) prior to photothrombotic stroke administration. Diet was mildly restricted to maintain approximately 90% of normal body weight. For the training, mice were placed in the plexiglass chamber with a narrow open slit window (14 × 1/2 inch), and 3.2 cm long pasta pieces were placed in a 5 × 5 orientation (pasta matrix). During the first week of training, mice were trained for 30 min/day with the pasta matrix set in front of the slit and tilted toward the chamber. During the second week, mice were trained in an upright pasta matrix for 30 min/day. During weeks 3 and 4, the training was conducted for 15 min/day, gradually moving the position of the pasta matrix to the left side of the mouse so that the entire pasta matrix was eventually positioned behind the chamber wall. In this way, most mice can reach the pasta with their right forelimb, which is impaired by the stroke. Motor performance was analyzed by counting the number of pasta pieces broken in the matrix as points and normalizing by the pre-stroke baseline value. We analyzed only the mice that scored at least 7 points on the baseline test and decreased at least 2 points after stroke to sensitively assess motor impairment and recovery.

#### Grid Walk Test

To assess innate motor control, mice were placed on mesh wire grids for 5 min. Each 5-min session was video recorded, and the number of right forelimb footfalls in the first 50 steps was counted. If the total number of steps was less than 50, all steps taken during the test were analyzed.

#### Tape Removal Test

An adhesive tape removal test was conducted to assess perceptual-motor control. A 4 mm × 3 mm adhesive tape was placed on both forepaws of the mice prior to testing. Each paw received a different color of tape for differentiation (left paw-yellow, right paw-red). After the adhesive tape was placed on each paw, the mice were timed on how long it took them to remove the adhesive tape from both paws. Mice were habituated to the task once a week for 4 weeks before stroke surgery. Results from the last session before surgery were used for baseline motor performance.

### Immunohistochemistry

After completing the last behavioral testing, the animals were perfused transcardially with 0.1 M phosphate-buffered saline, followed by 4% paraformaldehyde. The brains were then removed, postfixed overnight in 4% paraformaldehyde, and cryoprotected in 30% sucrose solution. Using a cryostat, the brains were sectioned into parallel series of 40 µm thick sections, with a 280 µm interval (Leica CM 3050). These sections were stored at − 20 °C until the staining procedure.

For immunohistological staining, the sections were incubated in sodium citrate at 80 °C for 20 min, followed by a 10-min cooling period at room temperature in the same solution. Then, the sections were incubated in a blocking solution containing 5% donkey serum for 1 h and incubated with primary antibodies overnight at 4 °C. The primary antibodies used were GFAP (1:500, Thermo Fisher Scientific, cat #13–0300), NeuN (1:500, Abcam, cat # ab104225), and Iba1 (1:500, Abcam, cat #ab5076). After the primary antibody incubation, the sections were incubated with fluorescence dye-conjugated secondary antibodies (Jackson Immuno Research) for 1 h at room temperature.

### Image Analysis

#### Infarct Volume Measurement

**Fig. 2:**
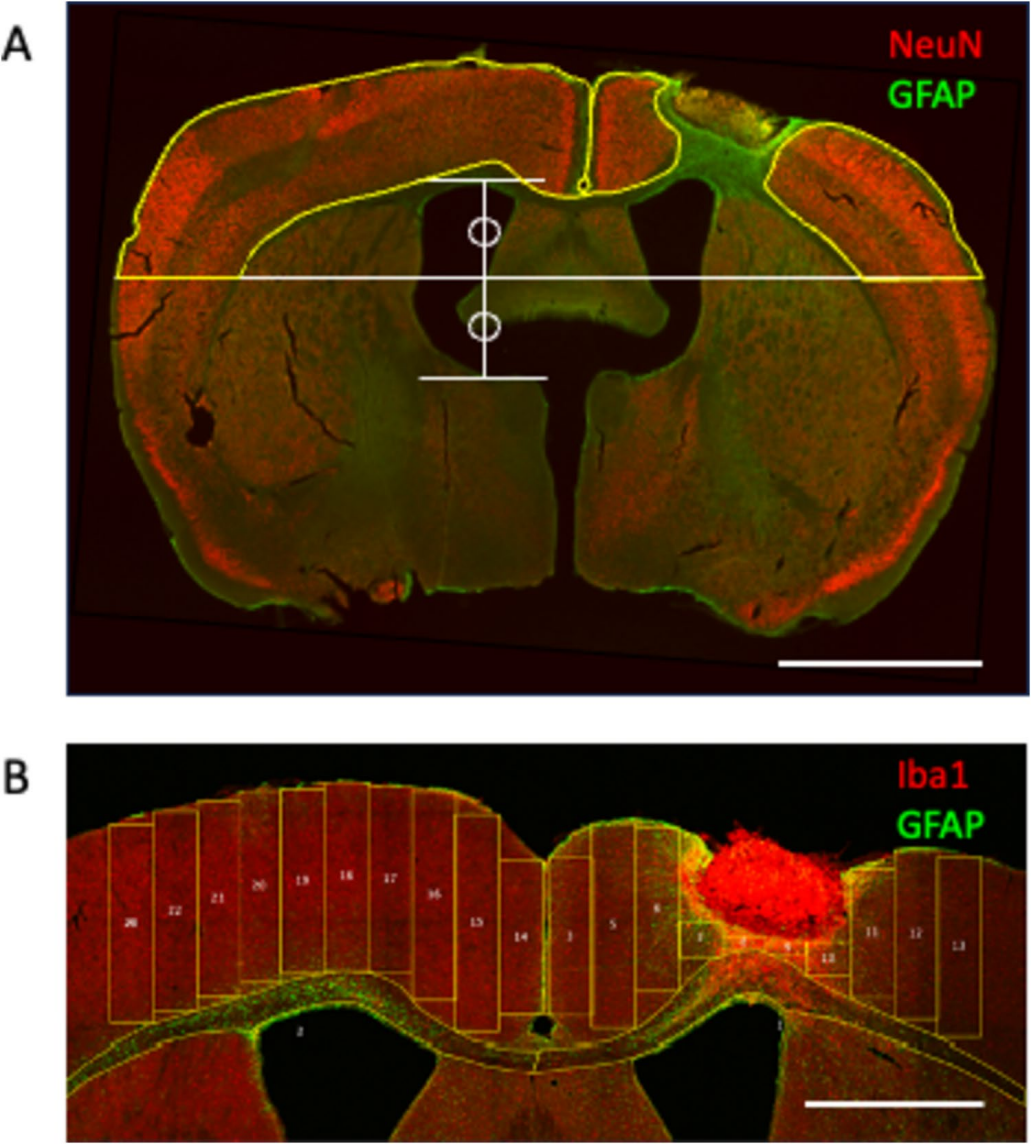
Image analysis for Infarct volume (**A**) and GFAP and Iba1 immunoreactivity (**B**). **A** The borders of the healthy cortical tissues were outlined. We analyzed the dorsal cortex determined by the center level of the ventricle. Scale bar, 2 mm. **B** Ten rectangle ROIs were set in the motor cortex, and immunofluorescence signals were measured. Scale bar, 1 mm

Serial brain sections with 280 µm intervals were stained with NeuN and GFAP antibodies, and images were taken using a confocal microscope (4 × objective; Nikon). The borders of intact dorsal cortical tissue were outlined with 10 brain sections covering the prefrontal cortex, motor cortex, and sensory cortex (1.5 to − 1.0 mm anterior to the bregma in MouseBrainAtlas). The ventral end of the dorsal cortical tissue was determined at the center level of the ventricle (Fig. 2A). The healthy tissue volume was calculated by multiplying the area and the section interval. The brain damage was calculated as follows:

$$Brain\;damage \left({mm}^{3}\right)=Contralesional\;cortex\;volume-Ipsilesional\;cortex\;volume$$

$$Brain\;damage \left(\mathrm{\%}\right)=\left(1-Ipsilesional\;cortex\;volume/Contralesional\;cortex\;volume\right)*100$$

#### Quantification of GFAP and Iba1 Immunoreactivity

After the immunohistochemistry with GFAP and Iba1 antibodies, z-stack images of the motor cortex were taken from the two sections with the most and the second most extensive infarct tissues using a confocal microscope (7 steps, 3 µm step interval, 20 × objective; Nikon). Using ImageJ software, maximum-intensity projection images were created. For white matter measurement, we outlined the white matter excluding the subventricular zone and measured the mean intensity of Iba1 and GFAP immunofluorescent signals. For cortical tissue measurement, we placed ten rectangle regions of interest (ROIs) with 246 µm width (100 pixels, 0.4051 pixel/µm) to fit in the cortical tissue excluding the infarct core, and measured the area and mean intensity of each ROI (Fig. 2B). Then, the mean intensity of 10 ROIs was calculated by the following formula.

$$Average\;intensity\; of\; 10\; ROIs = \sum (Mean\;intensity * Area) / \sum Area$$

These values were again averaged by two analyzed sections and further normalized by contralesional values.

### Statistical Analysis

All data are reported as mean ± standard deviation (SD). The normal distributions of all the data were tested by the Shapiro–Wilk test. Two-way repeated measure analysis of variance (ANOVA) was performed to compare behavioral measures across time points in the pasta matrix test and the grid walk test, confirming the normality of the data. For post hoc testing, Tukey’s multiple comparison tests were used. The data in the pasta matrix test were further analyzed with a linear mixed-effects model. Nonparametric ANOVA [54] was performed to analyze the data in the tape removal test, which does not follow normal distribution. For post hoc testing, the Kruskal–Wallis test was performed. We compared histological measurements using one-way ANOVA and Tukey’s multiple comparison tests. A statistical significance was defined as a *P* value < 0.05. Statistical analyses were carried out using Graph Pad Prism (Graphpad Software Inc.) and R (R Development Core Team).

## Results

### Number of Animals Analyzed and Excluded

A total of 72 mice were used. Fourteen mice that died after the stroke were excluded from the study. We also excluded four mice in which histological analysis did not demonstrate the presence of infarcted tissue. Accordingly, 54 mice were included in the study (2.5G/5–16-40 Hz: *n* = 9, 0.35G/5–16-40 Hz: *n* = 8, 2.5G/40 Hz: *n* = 10, 0.35G/40 Hz: *n* = 9, 0.35G/5 Hz: *n* = 10, 0G/0 Hz: *n* = 8).

### ELF-EMF Enhance Functional Recovery Depending on the Frequency/Intensity

**Fig. 3:**
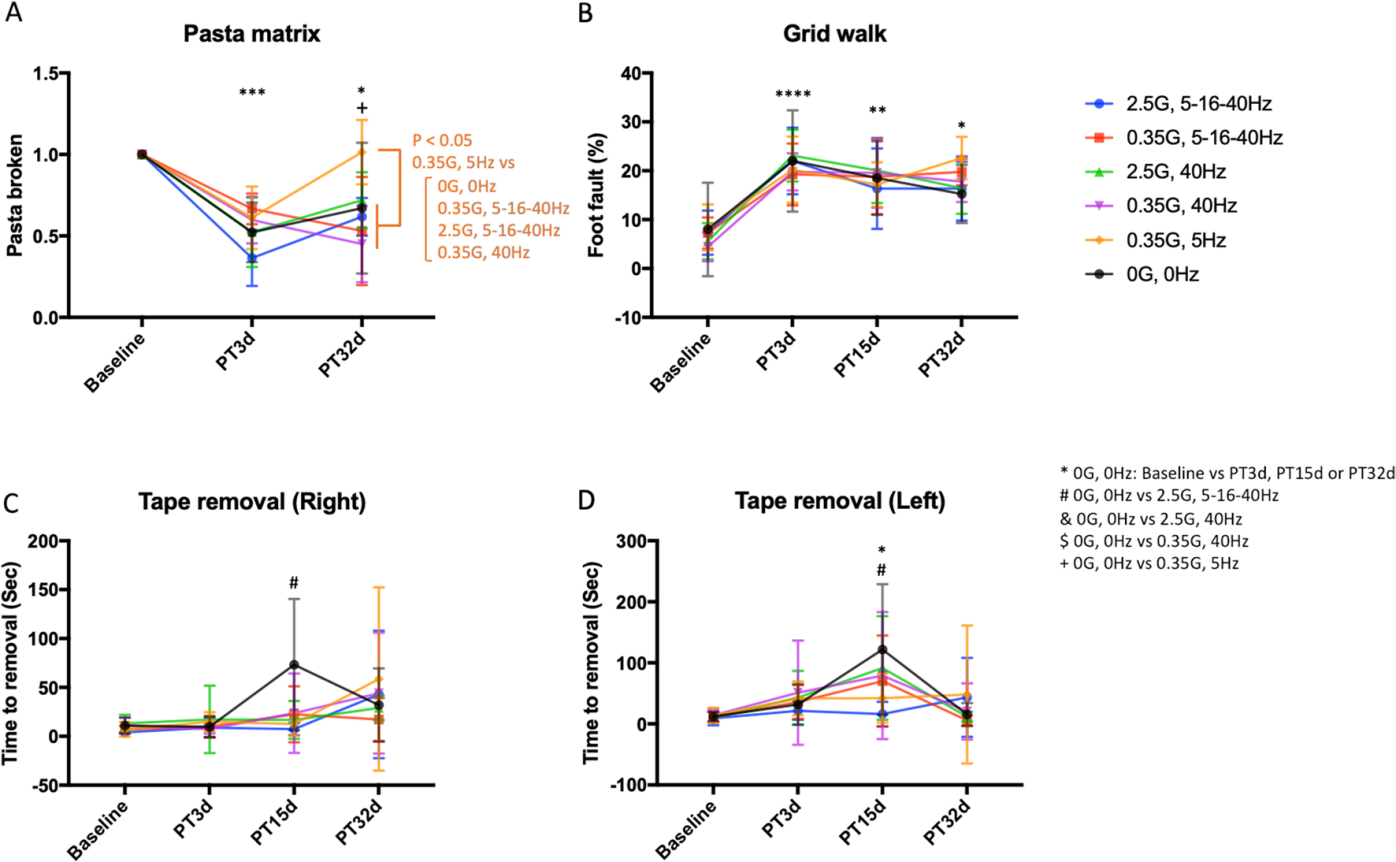
ELF-EMFs differently influence motor recovery depending on intensity-frequency parameters. Motor performance in the pasta matrix test (**A**), the grid walk test (**B**), and the tape removal test (**C** right forelimb, which is impaired by the stroke and **D** left, contralateral forelimb). Statistical significance at each time point compared with sham stimulation, 0G/0 Hz, is indicated by an asterisk. Data points represent mean ± SD. *P < 0.05, **P < 0.01, ***P < 0.001: 0G, 0 Hz baseline vs PT3d, PT15d, or PT32d, ##*P* < 0.01: 0G, 0 Hz vs 2.5G, 5–16-40 Hz, &&*P* < 0.01: 0G, 0 Hz vs 2.5G, 40 Hz, + *P* < 0.05, +  + *P* < 0.01: 0G, 0 Hz vs 0.35G, 5 Hz

The effects of ELF-EMF on functional recovery were analyzed in the mice after photothrombotic stroke using three motor tests, including the pasta matrix test, grid walk test, and adhesive tape removal test. In the pasta matrix test, mice in all the groups showed motor impairment 3 days after the stroke, followed by various degrees of recovery 32 days after the stroke (Fig. 3A two-way repeated measure ANOVA; time: *F*_2,50_ = 54.92, *P* < 0.0001, group: *F*_5,25_ = 3.157, *P* = 0.0241, interaction: *F*_10,50_ = 3.325, *P* = 0.0022). The sham stimulation group (0G/0 Hz) showed persistent disability through the experiment (Tukey’s multiple comparison tests, baseline vs PT3d; *P* = 0.0008, baseline vs PT32d; *P* = 0.0261). In contrast, we observed significant improvements with two ELF-EMF protocols, 2.5G/5–16-40 Hz, and 0.35G/5 Hz, during the recovery period (Tukey’s multiple comparison tests, PT3d vs PT32d; 2.5G/5–16-40 Hz: *P* = 0.0215, 0.35G/5 Hz: *P* = 0.0006). The improved motor performance was also evident in the 0.35G/5 Hz stimulation compared with the sham treatment for the final outcome measurement (Tukey’s multiple comparison tests, 0.35G/5 Hz vs 0G/0 Hz: PT32d: *P* = 0.0361), indicating enhanced functional recovery after ELF-EMF with this protocol.

Photothrombotic stroke caused severe impairment in the grid walk test. Similar to the pasta matrix test, the sham stimulation group showed persistent disability until the end of the study (Fig. 3B two-way repeated measure ANOVA; time: *F*_3,132_ = 57.6, *P* < 0.0001, Tukey’s multiple comparison tests, baseline vs PT3d; *P* < 0.0001, baseline vs PT15d; *P* = 0.0012, baseline vs PT32d; *P* = 0.0371). The recovery differences across groups were not significant (Group: *F*_5,44_ = 0.3220, *P* = 0.08971, interaction: *F*_15,132_ = 1.075, *P* = 0.3854). Although mice showed slight reductions in foot fault rate during the recovery period, these changes were not statistically significant in any stimulation conditions (Tukey’s multiple comparison tests, PT3d vs PT32d: *P* > 0.05). The tape removal test did not show significant impairment 3 days after the stroke, but delayed abnormalities were observed after the sham treatment in the left paws 15 days after the stroke (Fig. 3C and D non-parametric ANOVA; right-hand, time: WTS statistic = 20.53897, df = 3, *P* = 0.00013, group: WTS statistic = 19.56589, df = 5, *P* = 0.0015: left-hand, time: WTS statistic = 54.98333, df = 3, *P* < 0.0001, group: WTS statistic = 7.19202, df = 5, *P* = 0.20674, interaction: WTS statistic = 66.60031, df = 15, *P* < 0.0001, Kruskal–Wallis test, baseline vs PT15d: 0G/0 Hz, right-hand *P* = 0.1092, 0G/0 Hz, left-hand *P* = 0.045). The post hoc test also revealed a significant difference in the 0G/0 Hz group compared to the 2.5G/5–16-40 Hz group (Kruskal–Wallis test, *P* < 0.05).

Although the repeated-measures ANOVA is often used to analyze data collected in a repeated-measures study design, multilevel modeling techniques are more informative than ANOVA because they characterize both group-level (nomothetic) and individual-level (idiographic) effects, yielding a more complete understanding of the phenomena under study [55]. Thus, we further analyzed the results in the pasta matrix test using linear mixed model (LMM) analysis to better understand the effect of different ELF-EMF protocols. We fit LMM with “group” (the ELF-EMF protocol), “time” (days post-stroke), and their interactions as fixed effects to analyze “performance” (the number of pasta pieces broken). Additionally, we incorporated individual variations for each mouse as random effects. The LMM revealed that while group effects were not significant (vs 0G/0 Hz: 2.5G/4–16-40 Hz: *t* = 0.000, *P* = 1.000, 0.35G/5–16-40 Hz: *t* = 0.000, *P* = 1.000, 2.5G/40 Hz: *t* = 0.000, *P* = 1.000, 0.35G/40 Hz: *t* = 0.000, *P* = 1.000, 0.35G/5 Hz: *t* = 0.000, *P* = 1.000), time significantly affected motor performance (vs baseline; Day 3: *t* =  − 3.897, *P* = 0.00029, Day 32: *t* =  − 2.686, *P* = 0.0098), indicating sustained motor impairment in this motor task. Noteworthy, the interaction of group and time was significant only in the 0.35G/5 Hz*Day 32 (*t* = 2.172, *P* = 0.0346). This result suggests that 0.35G/5 Hz ELF-EMF may influence the performance differently after the treatment. Then, we explored the performance change in the recovery phase (Day 3 and 32) in each group using LMM. In this analysis, we fit the LMM with “time” (Day 3 and 32) as fixed effects and individual variations as random effects on “Performance” in each group. This analysis showed that time affected performance only in 2.5G/4–16-40 Hz and 0.35G/5 Hz groups (Day 3 vs Day 32: 0G/0 Hz: *t* = 0.779, *P* = 0.4928, 2.5G/4–16-40 Hz: *t* = 3.867, *P* = 0.01804, 0.35G/5–16-40 Hz: *t* =  − 0.882, *P* = 0.4277, 2.5G/40 Hz: *t* = 2.963, *P* = 0.0594, 0.35G/40 Hz: *t* =  − 1.422, *P* = 0.181, 0.35G/5 Hz: *t* = 3.587, *P* = 0.00495). These data indicate that improvement of motor performance depends on the ELF-EMF protocols. Finally, we computed the effect size of each treatment in the recovery phase to estimate the functional gains. Consistent with LMM analysis, 2.5G/4–16-40 Hz and 0.35G/5 Hz groups demonstrated the larger effect size than the other groups (Cohen’s d; d estimate: 0G/0 Hz: 0.477 ± 0.311, 2.5G/4–16-40 Hz: 2.040 ± 0.148, 0.35G/5–16-40 Hz: − 0.557 ± 0.244, 2.5G/40 Hz: 1.016 ± 0.193, 0.35G/40 Hz: − 0.759 ± 0.1945, 0.35G/5 Hz: 2.070 ± 0.194). These results indicate that the 2.5G/4–16-40 Hz and 0.35G/5 Hz cause better functional recovery in the forelimb function.

### ELF-EMF Does Not Affect Infarct Volume, Astrocyte activation, or Phagocytic Response After Stroke

ELF-EMFs influence various biological processes, including neuroprotection and neuroplasticity. Among these biological processes, cell death and tissue damage responses, particularly oxidative stress and inflammation, are relatively well-investigated effects induced by ELF-EMF. Thus, we performed histological analyses to assess the effects of ELF-EMF on neuronal tissue damage and neuroinflammation.

**Fig. 4:**
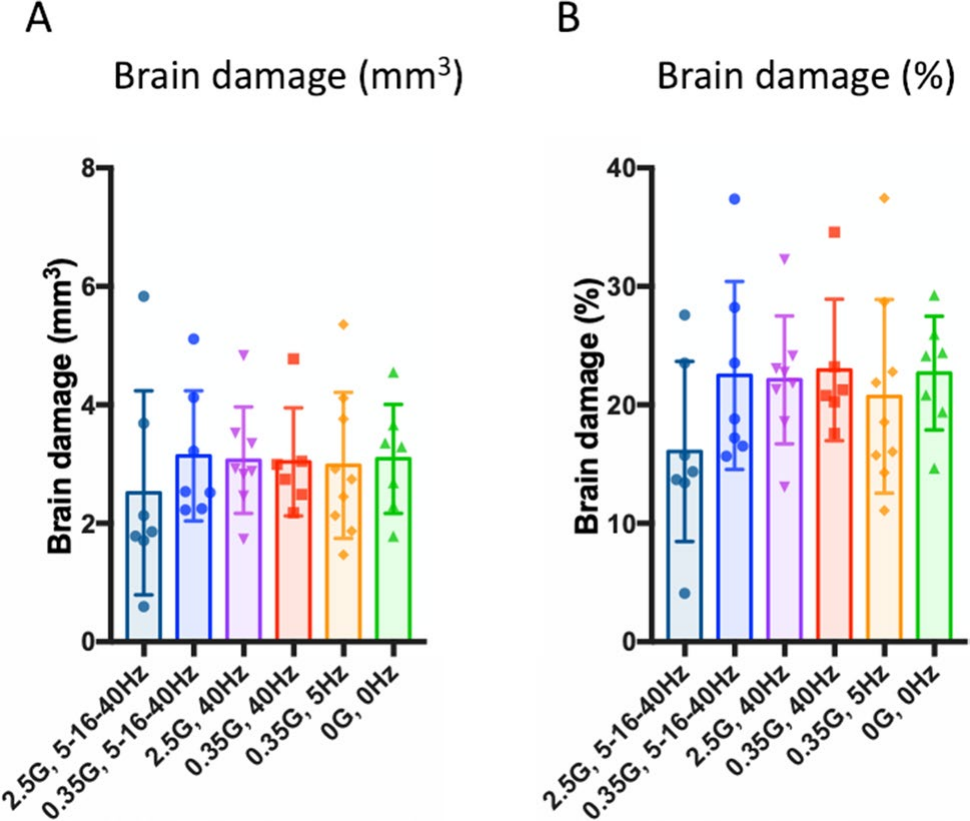
ELF-EMFs do not affect infarct volume. Healthy cortical tissue volumes were measured for the ipsilesional and contralesional cortex, and brain damage was calculated. No changes were observed in the brain damage across different ELF-EMF protocols. Data points represent mean ± SD

We first examined infarct volume after ELF-EMF stimulation. While we observed a slight reduction in brain damage after ELF-EMF stimulation with the 2.5G/5–15-40 Hz protocol, no significant change was detected in the brain damage measurement (Fig. 4A and B; one-way ANOVA: brain damage (mm^3^): *F*_5,38_ = 0.2717, *P* = 0.9258, brain damage (%): *F*_5,38_ = 1.015, *P* = 0.4223). These data indicate that ELF-EMF protocols used in the current study has no significant neuroprotective effect when initiated 4 days after stroke.

**Fig. 5:**
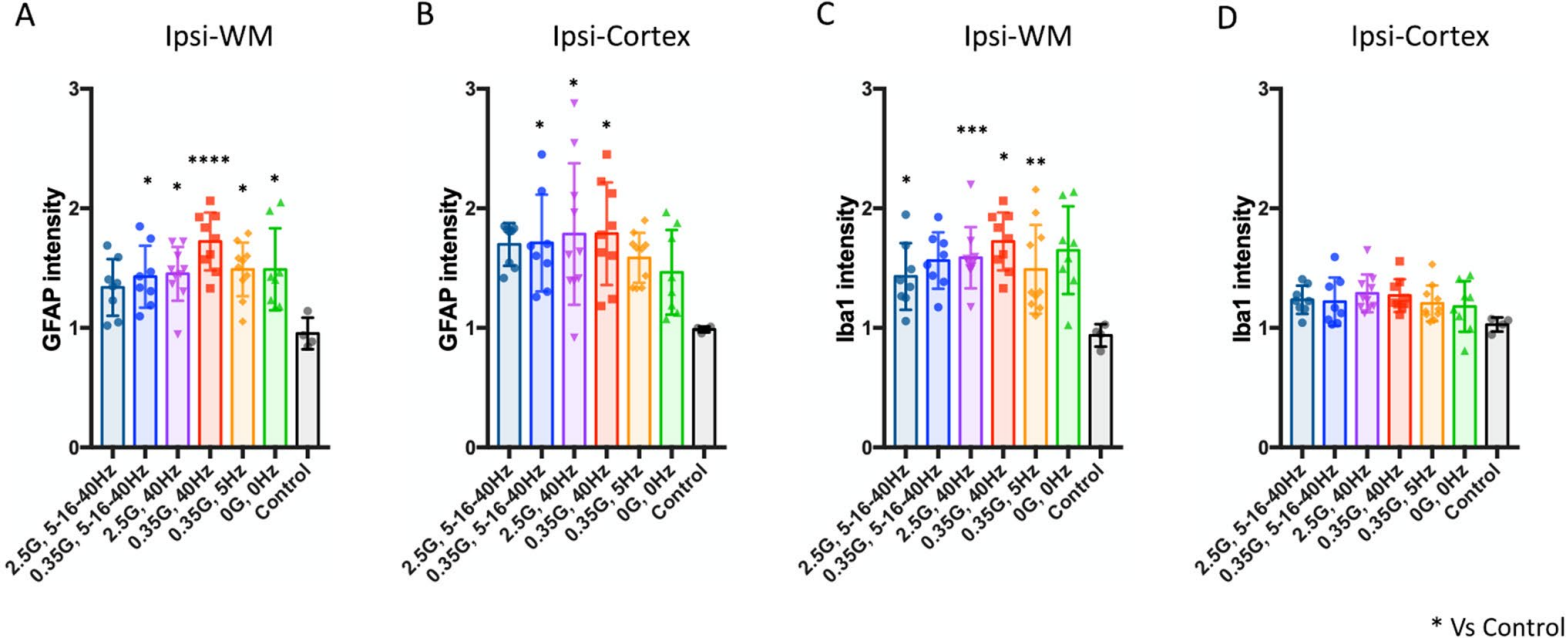
ELF-EMFs do not affect astrocyte and phagocyte activation. GFAP and Iba1 immunofluorescence signals were measured in ipsilesional and contralesional white matter and cortex. Ipsilesional signals were normalized by contralesional signals. Stroke increased GFAP signal in the white matter (**A**) and the cortex (**B**), and Iba1 signal in the white matter (**C**), but not in the cortex (**D**). No significant difference was observed between ELF-EMF groups. Data points represent mean ± SD

Subsequently, we examined the neuroinflammatory response mediated by astrocytes and phagocytes (microglia/macrophage). We observed an elevation in GFAP signals in both the ipsilesional white matter and cortex across all stimulation groups when compared to the control animals (Fig. 5A and B one-way ANOVA, Ipsi-WM: group: *F*_6,50_ = 4.784, *P* = 0.0006, Ispi-cortex: group: *F*_6,50_ = 2.810, *P* = 0.0196). Conversely, increased Iba1 signals were predominantly observed in the ipsilesional white matter, with subtle effects noted in the cortical tissues at the 32-day time point after stroke (Fig. 5C and D one-way ANOVA, Ipsi-WM: group: *F*_6,50_ = 3.945, *P* = 0.0026, Ispi-cortex: group: *F*_6,50_ = 1.524, *P* = 0.1896). While notable variations were observed among different stimulation conditions when compared to non-stroke control animals, no statistically significant differences were found in comparison with any ELF-EMF conditions and the sham stimulation. These data indicate that these specific ELF-EMF protocols do not significantly influence this aspect of the glial response after stroke.

## Discussion

In the present study, we investigated the effects of ELF-EMF on functional recovery beginning 4 days after stroke using various frequency-intensity protocols. Our behavioral analysis observed that ELF-EMFs had a distinctive impact depending on the intensity/frequency protocol, specifically on the pasta matrix test. Our comprehensive statistical analysis, utilizing LMM and ANOVA, revealed several key findings in this test: 1) The 0.35G/5 Hz ELF-EMF group showed significantly superior final outcomes compared to all other groups except the 2.5G/40 Hz (ANOVA and Tukey's post hoc test). 2) Only the 0.35G/5 Hz ELF-EMF group showed a significant time*group interaction (LMM). 3) The 0.35G/5 Hz and 2.5G/5–16-40 Hz ELF-EMF groups induced significant motor performance improvement during the ELF-EMFs treatment (LMM). 4) The functional gains achieved with ELF-EMFs were more pronounced in the 0.35G/5 Hz and 2.5G/5–16-40 Hz groups surpassing the other groups (Effect size analysis). These statistical findings strongly support the superior restorative effects of 0.35G/5 Hz ELF-EMF in the learned motor performance assessed by the pasta matrix test. Furthermore, the histological analysis revealed that ELF-EMF treatments had no significant impact on cortical tissue volume loss or astrocyte and phagocyte activation.

Considering the previous literature, five preclinical studies utilizing rodent stroke models have explored the effects of ELF-EMFs, employing various protocols as outlined in Table 2.

**Table 2:** Reference of the studies investigating ELF-EMFs using a rodent stroke model

	Year	Frequency	Intensity		Initiation time	Time/day	Treatment period	Animal	Stroke model	ELF-EMS effects
Pena-Philippides	2014	27.12 Hz × 2 Hz burst	3 V/m	0.1 mG	3–45 min	15 min × 2/day	21 days	Mouse	MCAO	Infarct volume
Segal	2016	3.93 Hz, 15.72 Hz	50 uT	0.5 G	2 d	2 min/day	every other day, 4 weeks	Rat	MCAO	Edema, motor function
Gao	2019	50 Hz	1 mT	10 G	1 d	2 h/day	28 days	Rat	MCAO	Neurogenesis, cognition
Font	2019	10, 60 Hz	13.5 mT	135 G	3 h	20 min/day	4 days	Rat	MCAO	Infarct volume, NO
Kemps	2022	60 Hz	13.5 mT	135 G	1 h	20 min/day	4 days	Mouse	MCAO	Infarct volume, CBF

While all of these studies utilized the middle cerebral artery occlusion (MCAO) model of rodent stroke, there were differences in frequency, intensity, initiation time, treatment duration, and overall treatment period for ELF-EMF application. Notably, the initiation time of treatment is a critical factor to consider. In our study, we initiated ELF-EMF treatment 4 days after stroke induction, which represents the latest time point among the reviewed studies. Neuronal death and brain infarct progression occur through sequential biological cascades, with the potential to rescue the peri-infarct core diminishing after approximately 48 h [56]. Previous studies demonstrated reduced infarct volume when ELF-EMF treatment was initiated within 3 h of stroke induction. In contrast, our study focused on the effect of ELF-EMF in the subacute phase, in which the initial tissue damage subsides. Our histological analysis confirmed that ELF-EMF treatment initiated 4 days after stroke did not significantly affect infarct volume or glial activation, providing evidence of minimal impact on neuronal protection.

Interestingly, we observed that theta-frequency ELF-EMFs (5 Hz) demonstrated greater efficacy in restoring motor function compared to gamma-frequency ELF-EMFs (40 Hz), when histological indicators of neuroprotection tested here were minimal. Previous studies focusing on neuroprotection have reported that gamma-frequency ELF-EMF are more effective than theta-frequency ELF-EMF [31]. This discrepancy suggests that the optimal frequency may vary depending on whether the targeting mechanism is neuroprotection during the acute phase or biological processes during the subacute and chronic phases. One possible target in the subacute and chronic phase would be the promotion of neuroplasticity. Dufor and colleagues reported that low-intensity magnetic stimulation with either theta burst or biomimetic high-frequency pattern induces axon outgrowth and synaptogenesis after olivocerebellar lesioning via magnetoreceptors [21]. In addition to neuronal plasticity, blood flow modulation is another potential target. ELF-EMFs modulate nitric oxide signaling, which regulates cerebral blood flow in both acute (vascular tone) and chronic phases of stroke (angiogenesis) [57]. While nitric oxide signaling is speculated as a mechanism of neuroprotection by ELF-EMFs in the acute phase [26], it may also induce beneficial effects on tissue repair in the subacute phase. Although improved functional recovery without significant neuroprotective effects after the ELF-EMFs suggest the involvement of neuronal repair or plasticity, the present study did not directly investigate them. Therefore, observation of plasticity markers, such as axonal remodeling, synaptogenesis, neurogenesis, and angiogenesis, and further mechanical studies, including loss of function studies, are warranted.

Recent studies have highlighted the significance of low-frequency oscillatory activity for skilled upper-limb tasks in stroke recovery [58, 59], suggesting that low-frequency ELF-EMF may modulate such oscillatory activity. More specifically, in a recent clinical study, results showcase the efficacy of motor-related oscillations in hastening recovery when initiated during the subacute phase following a stroke [33]. Furthermore, the comprehensive multicenter study, EMAGINE [60], underscores the importance of understanding the fundamental mechanism through which ELF-EMF operates, an issue addressed by the current findings.

We should note that the motor tests used in this study assessed different skills and abilities. We observed sustained motor impairment in the pasta matrix test and the grid walk test but not in the adhesive tape removal test, which is generally considered a sensorimotor test and is susceptible to compensatory strategies [61]. Since our stroke model targeted only the forelimb motor area, preserved motor and sensory areas may have contributed to a quick recovery in this task. Responsiveness to ELF-EMF also differed between the two behavioral tests in which impairment persisted. Functional recovery was strongly dependent on the ELF-EMF protocol in the pasta matrix test. In contrast, the recovery pattern was nearly identical in all groups in the grid walking test. While the grid walk test assesses innate motor function and limb coordination, the pasta matrix test assesses the ability to perform learned motor tasks that require skilled forelimb movements [62]. Recent studies have shown unique brain synchronization in skilled reaching behavior in both execution and learning processes [59, 63]. Additionally, parvalbumin interneurons, which play a pivotal role in neuronal synchronization, including gamma wave generation [64] and spike-phase coupling to theta oscillation [65], are activated in forelimb-reaching behavior. Therefore, ELF-EMF may stimulate the specific types of oscillation or neurons associated with skilled forelimb movements and lead to functional recovery.

The choice of stroke model is another crucial factor in data interpretation. We chose the photothrombotic stroke model to precisely induce a cortical lesion in the forelimb motor area. This model results in sustained motor impairments but relatively mild sensory impairment compared to the MCAO model, which primarily affects the striatum and sensory cortex rather than the motor cortex. These differences can influence motor impairment and recovery patterns post-stroke. While MCAO is advantageous for investigating neuroprotective effects due to its large penumbra region, the photothrombotic stroke model is valuable for understanding the recovery process or neuronal network reorganization, as it can target specific brain areas based on the function of interest. The choice of stroke model can also impact the effectiveness of ELF-EMFs. For instance, a prior rTMS study demonstrated that 10 Hz rTMS effectively improved movement kinematics in subcortical stroke patients but had no effect or worsened outcomes in cortical stroke patients [66]. These findings underscore the importance of optimizing parameters for each stroke type, and preclinical research involving multiple stroke models can provide valuable insights in this regard.

It is noteworthy that our study’s context includes the evolving landscape of stroke management. In the acute phase after stroke, recanalization therapies like endovascular therapy and intravenous thrombolysis are the gold standard. Non-invasive brain stimulation, such as ELF-EMF therapy, holds promise as an adjunctive therapy in cases where recanalization is not feasible, and it may also enhance tissue repair processes. Therefore, the development of effective ELF-EMF protocols for later time points in the post-stroke recovery period could substantially augment the value of this therapeutic approach. Recent STAIR recommendations also suggest that elements of the neurovascular unit—neurons, astrocytes, endothelial cells, and pericytes—exhibit different vulnerabilities that evolve on different time scales in different brain regions and propose that treatments can be developed to target different elements of the neurovascular unit at different times [67]. These ideas strongly support the rationale of the current study.

In the current study, we experienced a relatively high mortality rate after stroke. Although we conducted a thorough retrospective analysis of the animals’ general health and surgery records, we were unable to identify the specific reason behind the elevated mortality rate. Since we placed a strong emphasis on investigating motor impairment in learned motor performance, which necessitated a rigorous and prolonged behavioral training regimen involving pasta matrix training over a span of 5 weeks, accompanied by moderate food restriction. While we took every precaution to ensure the animals’ well-being during this training, the extended and demanding nature of the regimen might have induced stress, potentially contributing to the increased mortality rate observed. Therefore, more precautions must be taken for experimental design to minimize potential stress caused by behavior training in future studies.

In summary, our study demonstrated the positive effects of 0.35G/5 Hz ELF-EMFs on functional recovery in the learned motor performance without significant impact on cortical tissue volume loss or glial activation. The optimal frequency for therapeutic application may differ depending on the stage of stroke and the targeted biological process. Further research is necessary to explore the optimal parameters and long-term effects of ELF-EMF treatment for stroke rehabilitation.

## Data Availability

The supporting data of this study are available on request from the corresponding author.
